# Bedside Lung Ultrasound for Early Postoperative Risk Stratification for Prolonged Mechanical Ventilation After Liver Transplantation

**DOI:** 10.3390/jcm15208100

**Published:** 2026-10-19

**Authors:** Hui Wang, Yongyi Chen, Dongdong Han, Jun Duan

**Affiliations:** 1 Intensive Care Unit, China-Japan Friendship Hospital, 2 Yinghua Dongjie, Beijing 100029, China; wanghui@cjfh.org.cn (H.W.); chenyongyi@cjfh.org.cn (Y.C.); 2 Department of General Surgery, China-Japan Friendship Hospital, 2 Yinghua Dongjie, Beijing 100029, China; surghandd@163.com

**Keywords:** lung ultrasound, liver transplantation, mechanical ventilation, postoperative pulmonary complications, risk prediction

## Abstract

**Objectives:** Pulmonary complications remain a major determinant of postoperative morbidity following liver transplantation and frequently lead to prolonged mechanical ventilation (PMV). This study aimed to evaluate the value of lung ultrasound (LUS) for early postoperative risk stratification of PMV and to develop a multivariable model integrating LUS with conventional clinical variables. **Methods:** In this prospective cohort study, 116 adults undergoing primary liver transplantation were enrolled. A standardized 12-zone LUS examination was performed within 1–3 h of ICU admission. PMV was defined as the need for mechanical ventilation for ≥24 h. Multivariable logistic regression analysis was constructed by sequentially incorporating perioperative variables and postoperative PaO_2_/FiO_2_, followed by LUS. Model performance was evaluated using discrimination, calibration, bootstrap internal validation, and decision curve analysis. **Results:** PMV occurred in 39.7% (*n* = 46) of the patients. Those requiring PMV had significantly higher early postoperative LUS scores than patients extubated within 24 h (median: 18.0 vs. 12.0, *p* < 0.001). LUS scores were inversely correlated with oxygenation (SpO_2_/FiO_2_ ratio, ⍴ = −0.615, *p* < 0.001). LUS remained independently associated with PMV (OR = 1.52, 95% CI: 1.27–1.83). LUS alone demonstrated good discrimination (AUC: 0.830). After postoperative PaO_2_/FiO_2_ was included in the clinical model, incorporation of LUS significantly improved model discrimination (AUC: 0.935, 95% CI: 0.894–0.976) compared with the model without LUS (AUC: 0.862, DeLong *p* = 0.011). Decision curve analysis showed greater net benefit of the LUS-integrated model across clinically relevant threshold probabilities. **Conclusions:** Early postoperative LUS is independently associated with prolonged mechanical ventilation after liver transplantation. Integration of LUS into a multivariable framework provides incremental prognostic information beyond conventional postoperative respiratory assessment and may improve early postoperative risk stratification. External validation is required before clinical implementation.

## 1. Introduction

Liver transplantation is the definitive treatment for end-stage liver disease, and advances in surgical techniques and perioperative management have markedly improved survival [1]. Nevertheless, postoperative pulmonary complications remain common and are a major contributor to early morbidity. Several perioperative factors, including prolonged operative duration, substantial transfusion or fluid transfusion, hemodynamic instability, and predisposition to systemic inflammatory responses, may contribute to postoperative respiratory dysfunction [2,3,4].

Pulmonary edema, atelectasis, pleural effusion, acute respiratory distress syndrome (ARDS), and pulmonary infection frequently occur in the early postoperative period. These complications impair oxygenation and often necessitate extended respiratory support, and thereby adversely affecting clinical recovery [2,5]. Although mechanical ventilation (MV) is often essential in the immediate postoperative period, prolonged ventilation is associated with ventilator-associated pneumonia, ventilator-induced lung injury, and extended ICU stays [6]. Timely identification of patients at risk for prolonged mechanical ventilation (PMV) is therefore clinically important, as it may allow earlier intervention and more tailored postoperative respiratory support.

Lung ultrasound (LUS) has gained increasing attention in perioperative and critical care practice as a bedside imaging modality for the assessment of pulmonary aeration. Compared with chest radiography, LUS provides greater sensitive in detecting interstitial edema and pleural abnormalities and enables bedside detection of peripheral consolidations extending to the pleural surface. The LUS score offers a semi-quantitative evaluation of lung aeration by integrating findings such as B-lines, peripheral consolidation, and pleural effusion [7]. Previous studies have demonstrated the utility of LUS scoring in assessing the severity of pulmonary dysfuction and predicting respiratory outcomes, including the need for PMV [8,9,10]. However, the role of bedside LUS in early postoperative risk stratification for PMV after liver transplantation remains incompletely understood.

In this prospective cohort study, we sought to evaluate the association between early postoperative LUS scores and PMV after liver transplantation. We further investigated whether adding LUS to a multivariable clinical model improved risk discrimination beyond conventional perioperative variables.

## 2. Materials and Methods

### 2.1. Study Design

This prospective cohort study was conducted in the intensive care unit (ICU) of China–Japan Friendship Hospital between November 2022 and May 2024. Consecutive adult patients (≥18 years) undergoing primary liver transplantation and requiring postoperative mechanical ventilation with ICU admission were screened for eligibility. Exclusion criteria were: (1) multi-organ transplantation or repeat liver transplantation; (2) pre-existing end-stage pulmonary disease, defined as documented severe and irreversible pulmonary dysfunction despite optimal medical therapy, with chronic respiratory failure characterized by resting hypoxemia and/or hypercapnia and substantial functional limitation; (3) mechanical ventilation before surgery; (4) death within 48 h postoperatively due to causes other than primary respiratory failure; and (5) refusal to participate.

This study was approved by the Institutional Review Board of China–Japan Friendship Hospital (Approval No. 2022-KY-202). All methods were carried out in accordance with relevant guidelines and regulations. Written informed consent was obtained from all subjects or their legal representatives. To ensure patient privacy, all personal identifying information, including names and hospital identifiers, was removed from the dataset and manuscript.

Upon ICU admission, all patients were managed with standardized mechanical ventilation settings, including a tidal volume of 7–8 mL/kg predicted body weight and positive end-expiratory pressure (PEEP) of 5–10 cmH_2_O.

Patients were followed from ICU admission until hospital discharge or in-hospital death.

No formal sample size calculation was performed. The study size was determined by the number of eligible patients during the study period. The number of outcome events was considered sufficient to support multivariable regression analysis according to recommended events-per-variable criteria.

### 2.2. Data Collection

Clinical and outcome data were extracted from the electronic medical record system using standardized definitions. Potential confounders were selected a priori based on clinical relevance and the previous literature.

### 2.3. Ultrasound Assessment

Cardiopulmonary ultrasound examinations were performed within 1–3 h after ICU admission, once hemodynamic stability had been achieved. All examinations were conducted using a CX-50 ultrasound system (Philips Ultrasound Inc., Bothell, WA, USA) equipped with a curvilinear transducer (2–5 MHz). A standardized 12-zone protocol was applied, including two anterior, two lateral, and two posterolateral regions. Posterior lung regions were assessed by gently rotating the patient laterally to each side with assistance. Each predefined lung region was systematically scanned across the accessible intercostal spaces rather than assessed at a single fixed point. When different aeration patterns were observed within the same region, the regional LUS score was assigned according to the most severe ultrasound pattern identified. Lung aeration patterns were classified as follows [11]: N, normal aeration, defined as lung sliding with A-lines or fewer than three isolated B-lines; B1, moderate loss of lung aeration, characterized by multiple well-defined B-lines; B2, severe loss of lung aeration, characterized by multiple coalescent B-lines; and C, peripheral consolidation, characterized by tissue-like pattern with or without air bronchograms. Each zone was scored as: N-0; B1-1; B2-2, and C-3. The total LUS scores ranged from 0 to 36 points. For descriptive analysis, lung aeration loss was categorized into low (0–11), intermediate (12–15), and high (16–36) LUS groups according to tertiles of LUS score distribution in the present cohort. These categories were used for exploratory descriptive comparisons and were not intended to represent externally validated clinical severity thresholds.

Cardiac ultrasound was performed using the apical four-chamber view. Aortic flow was assessed by pulsed-wave Doppler, with the sample volume positioned at the left ventricular outflow tract (LVOT) to measure the velocity–time integral (VTI). Transmitral inflow was evaluated by pulsed Doppler with the sample volume placed at the mitral leaflet tips, allowing measurement of early (E) and late (A) diastolic peak velocities. Tissue Doppler imaging was used to assess mitral annular velocities, with a 5 mm sample volume positioned at the lateral mitral annulus to obtain early diastolic velocity (Ea). Transmitral E/A ratio and the lateral E/Ea ratios were measured as conventional echocardiographic indices of left ventricular diastolic function, with E/Ea used as an indirect marker of left ventricular filling pressure [12]. Inferior vena cava (IVC) assessment was not routinely used for volume evaluation, as its reliability may be limited during orthotopic liver transplantation due to surgical and hemodynamic alterations.

All ultrasound examinations were performed by two experienced intensivists who were blinded to subsequent clinical outcomes and to each other’s assessments. Because the examinations were performed at the bedside in the ICU, complete blinding to contemporaneous clinical information and ventilator settings was not feasible; however, the operators did not have access to the study outcome data at the time of LUS assessment. All examinations and LUS scoring were performed according to a predefined standardized protocol. Interobserver agreement was evaluated in a randomly selected subset of patients.

### 2.4. Clinical Parameters

Intraoperative and early postoperative variables were systematically recorded. These included operative duration, cumulative fluid balance, and clinical parameters at ICU admission, such as serum lactate concentration, requirement for vasoactive support, and MV settings (tidal volume [VT], PEEP, PaO_2_/FiO_2_ and SpO_2_/FiO_2_ ratios). Disease severity at ICU admission was assessed using the Acute Physiology and Chronic Health Evaluation II (APACHE II) score. Baseline liver disease severity before transplantation was evaluated using the model for end-stage liver disease (MELD) score.

### 2.5. Definition of PMV

The primary outcome was PMV, defined as invasive mechanical ventilation lasting ≥24 h after liver transplantation. This threshold was selected because early extubation within the first 24 postoperative hours is an established goal of contemporary liver transplantation pathways and has been widely adopted in previous liver transplantation studies [13]. Patients requiring reintubation within 48 h after extubation were also classified as having PMV. Outcome variables included duration of endotracheal intubation, total duration of mechanical ventilation, length of ICU stay, total hospital stay, and in-hospital mortality.

### 2.6. Statistical Analysis

Continuous variables were expressed as mean (standard deviation, SD) or median [interquartile range, IQR], as appropriate. Normality was assessed using the Shapiro–Wilk test. Duration of mechanical ventilation and length of ICU stay showed right-skewed distributions and were log_10_-transformed before parametric analyses to improve normality. Results are presented in original units for clinical interpretability. Categorical variables were expressed as counts and percentage. Missing data were handled using multiple imputation with chained equations. All candidate variables were included in the imputation model, and pooled estimates from the imputed datasets were used for subsequent analyses. Between-group comparisons were performed using the independent-sample t-test or Mann–Whitney U test for continuous variables and the χ^2^ test or Fisher’s exact test for categorical variables. One-way ANOVA or the Kruskal–Wallis test was used for comparisons across more than two groups. Spearman’s rank correlation was used to assess the association between LUS and oxygenation indices.

For regression analyses, continuous variables were retained in their original continuous form to preserve statistical information. Odd ratios (ORs) were expressed per one-point increase in LUS score. Variables with *p* < 0.10 in univariable analysis and those considered clinically relevant were entered into the multivariable logistic regression model. Multicollinearity was assessed using variance inflation factors. Model discrimination was evaluated using the area under the receiver operating characteristic curve (AUC), with comparisons performed using the DeLong test. Calibration was assessed using the Hosmer–Lemeshow test, calibration plots, calibration intercept, and calibration slope, and overall predictive performance was additionally assessed using the Brier score.

To evaluate the incremental predictive value of LUS, hierarchical logistic regression models were constructed according to the temporal availability of predictors. Model 1 included the preoperative MELD score; Model 2 additionally included intraoperative fluid balance; Model 3 further incorporated postoperative PaO_2_/FiO_2_; and Model 4 additionally included the LUS score. Changes in discrimination across the hierarchical models were evaluated using AUCs, with the incremental discrimination of Model 4 over Model 3 assessed using the DeLong test. Brier scores were used to assess overall predictive performance.

Internal validation was performed using 1000 bootstrap resamples to quantify model optimism. Optimism-corrected estimates of discrimination, Brier score, calibration intercept, and calibration slope were obtained. A calibration slope below 1 was interpreted as indicating some degree of model overfitting. To further assess model stability, bootstrap-based uniform coefficient shrinkage was performed using 1000 bootstrap resamples. The mean calibration slope obtained by applying each bootstrap-derived model to the original dataset was used as the uniform shrinkage factor. The original regression coefficients, excluding the intercept, were multiplied by this factor, after which the model intercept was re-estimated. Decision curve analysis (DCA) was performed to compare clinical net benefit of the clinical–physiological model (Model 3) and the model incorporating LUS (Model 4) across clinically relevant threshold probabilities.

As a sensitivity analysis addressing the potential influence of institutional extubation practices on the primary PMV definition, PMV was alternatively defined as mechanical ventilation lasting >48 h. Because 29 patients met this alternative endpoint, Firth’s penalized logistic regression was performed using the same four predictors included in the primary model (MELD score, intraoperative fluid balance, postoperative PaO_2_/FiO_2_, and LUS score). The discriminative performance of LUS for this alternative endpoint was additionally assessed using the AUC.

Interobserver agreement was assessed using Cohen’s kappa coefficient for regional LUS score and the intraclass correlation coefficient (two-way random-effects model, absolute agreement) for total LUS scores.

There was no loss to follow-up, as all patients were followed until hospital discharge or in-hospital death. All tests were two-sided, and a *p* value < 0.05 was considered statistically significant. Analyses were performed using SPSS version 25.0 (IBM Corp., Armonk, NY, USA) and R software (version 4.3.1; R Foundation for Statistical Computing, Vienna, Austria).

## 3. Results

### 3.1. Patients Enrollment and Study Population

**Figure 1:**
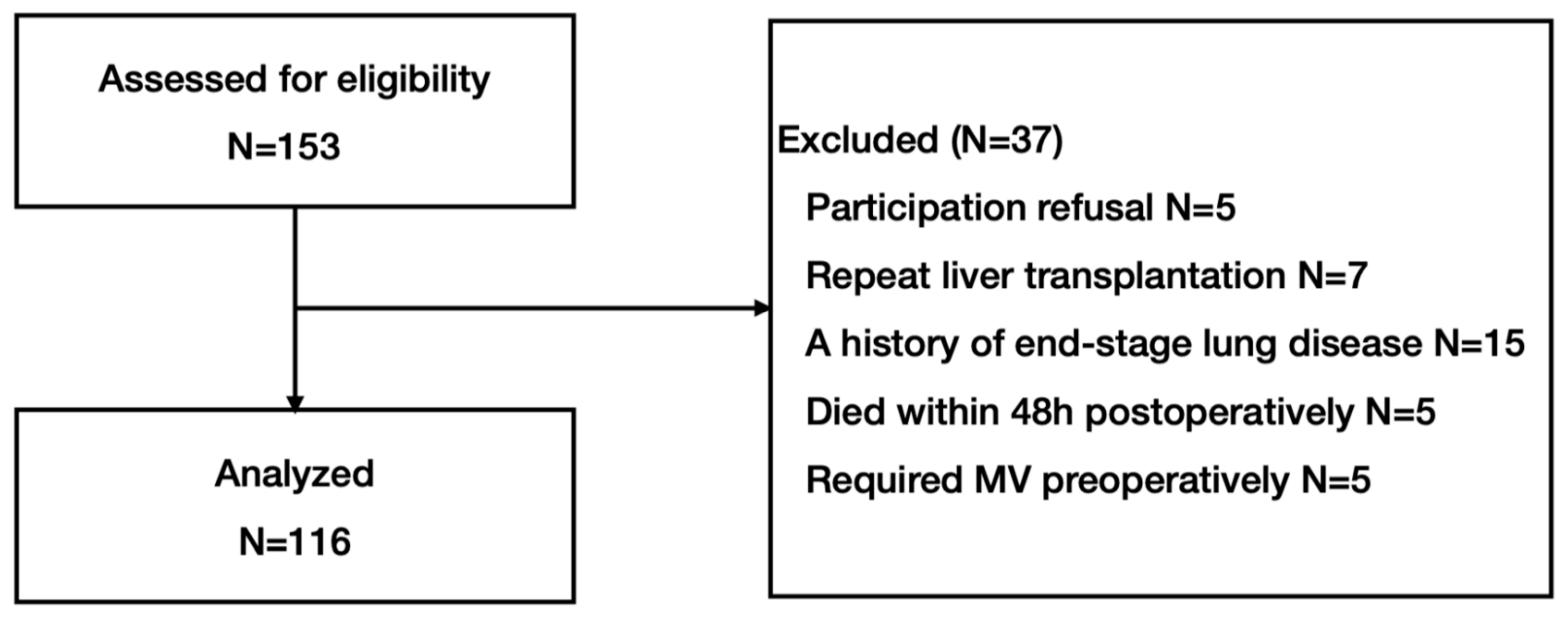
Patient recruitment flowchart. Flow diagram illustrating patient enrollment and reasons for exclusion. Numbers of included and excluded patients at each stage are shown, along with the final study population.

During the study period, 156 consecutive adult patients underwent liver transplantation and were screened for eligibility. A total of 37 patients were excluded for predefined reasons: refusal to participate (*n* = 5), repeat liver transplantation (*n* = 7), history of end-stage lung disease (*n* = 15), death within 48 h postoperatively (*n* = 5), and requirement for preoperative mechanical ventilation (*n* = 5). A total of 116 patients were included in the final analysis. All patients completed in-hospital follow-up until discharge or death, and no loss to follow-up occurred for the primary outcome. The study flow diagram is presented in Figure 1.

### 3.2. Baseline Characteristics

The baseline demographic and clinical characteristics of the study cohort are summarized in Table 1. The final cohort comprised 116 patients (85 males, 31 females) with a mean age of 52.23 (9.94) years. Patients were stratified according to the duration of invasive MV: <24 h (*n* = 70) and ≥24 h (*n* = 46). Chronic hepatitis B was the leading etiology of liver failure (41%), followed by autoimmune and alcoholic liver diseases (11% each).

Baseline demographics, including age, sex, body mass index, and smoking history, were comparable between the two groups (all *p* > 0.05). Similarly, no significant differences were observed in pre-existing comorbidities, intraoperative vasopressor use, or perioperative blood lactate levels.

However, patients requiring ≥24 h of MV demonstrated greater perioperative disease severity. They had significantly higher MELD scores [22.5 (14.8–29.5) vs. 12.7 (8.2–18.4)], longer operative durations [11.7 (2.6) vs. 9.7 (2.8) h] and greater intraoperative fluid balance [5.8 (4.1–9.0) vs. 4.7 (2.4–6.4) L] (all *p* < 0.01). Postoperatively, the ≥24 h MV group exhibited impaired oxygenation, reflected by lower PaO_2_/FiO_2_ and SpO_2_/FiO_2_ ratios, higher PEEP requirements and APACHE II scores.

Regarding clinical outcomes, patients with PMV experienced significantly longer ICU and hospital stays and higher in-hospital mortality (15% vs. 2.9%, *p* = 0.02). Lung ultrasound assessments demonstrated significantly higher LUS scores [18.0 (14.0–22.3) vs. 12.0 (8.0–14.0), *p* < 0.001]. The E/A ratios were also elevated in this group, whereas LVOT-VTI and E/Ea ratios did not differ significantly between groups.

Missing data were minimal. The proportion of missing values ranged from 0% to 3% across baseline variables, with the highest proportion observed for APACHE II score (2.5%).

### 3.3. Clinical Characteristics and Oxygenation Across LUS Tertiles

**Figure 2:**
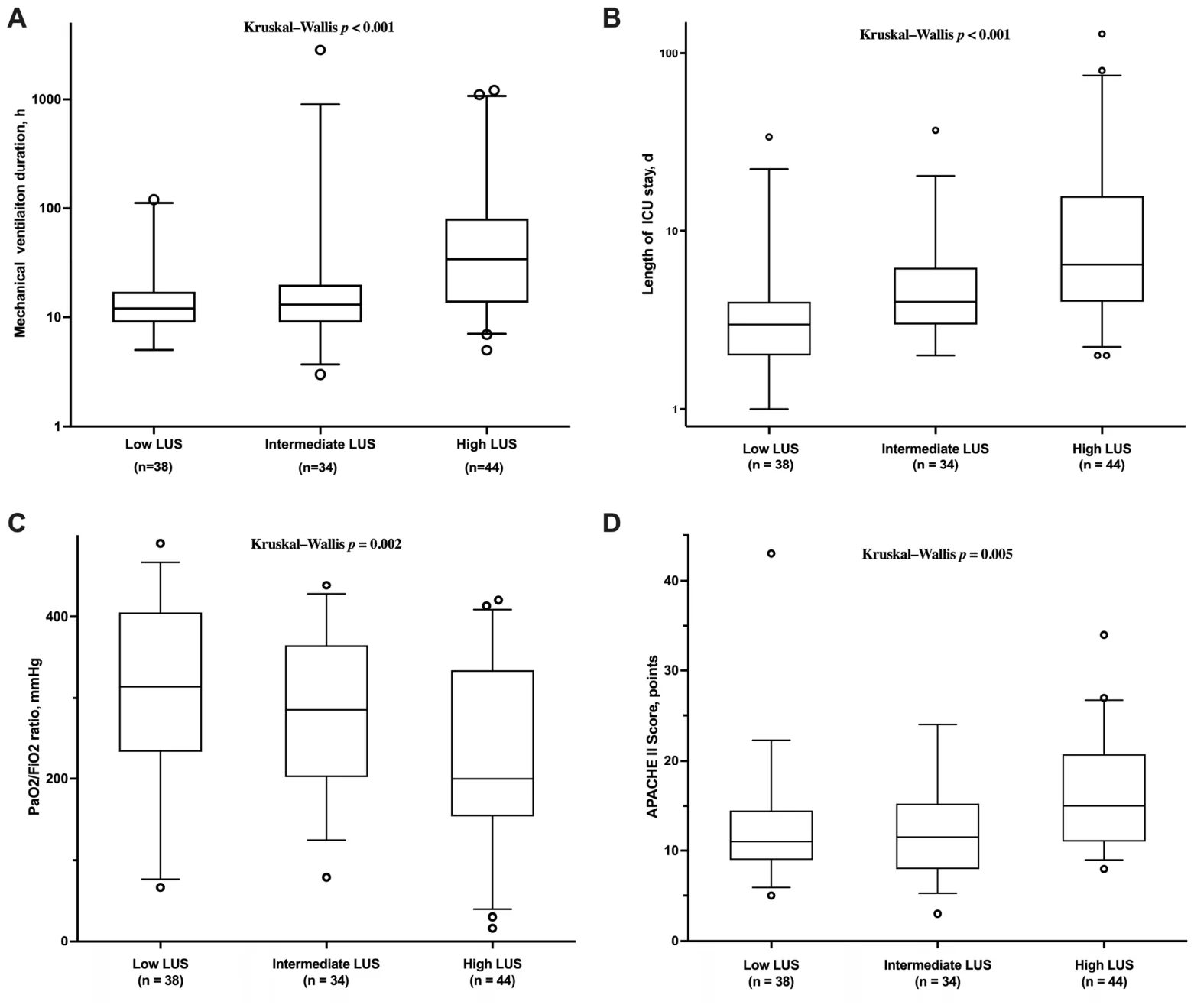
Clinical characteristics and short-term outcomes across cohort-derived LUS tertiles. Patients were stratified into low-, intermediate-, and high-LUS groups according to cohort-derived tertiles of the admission LUS score. Higher LUS scores were associated with longer duration of mechanical ventilation (**A**), prolonged length of ICU stay (**B**) (both log_10_-transformed), lower postoperative PaO_2_/FiO_2_ ratios (**C**), and higher APACHE II scores (**D**). Data are presented as box-and-whisker plots. The center line represents the median, the box represents the interquartile range (25th–75th percentiles), and the whiskers extend to the most extreme values within 1.5 times the interquartile range. Individual dots beyond the whiskers represent outliers. *p* values for overall group comparisons are presented.

Table 2 presents clinical outcomes stratified by LUS tertiles, reflecting increasing severity of lung aeration loss. Higher LUS categories were associated with progressively impaired oxygenation, as indicated by lower PaO_2_/FiO_2_ and SpO_2_/FiO_2_ ratios, along with higher APACHE II scores and longer durations of mechanical ventilation and ICU stay (all *p* < 0.05). These observations, illustrated in the box plots of Figure 2, indicate that greater loss of lung aeration corresponds to worsening clinical severity.

**Figure 3:**
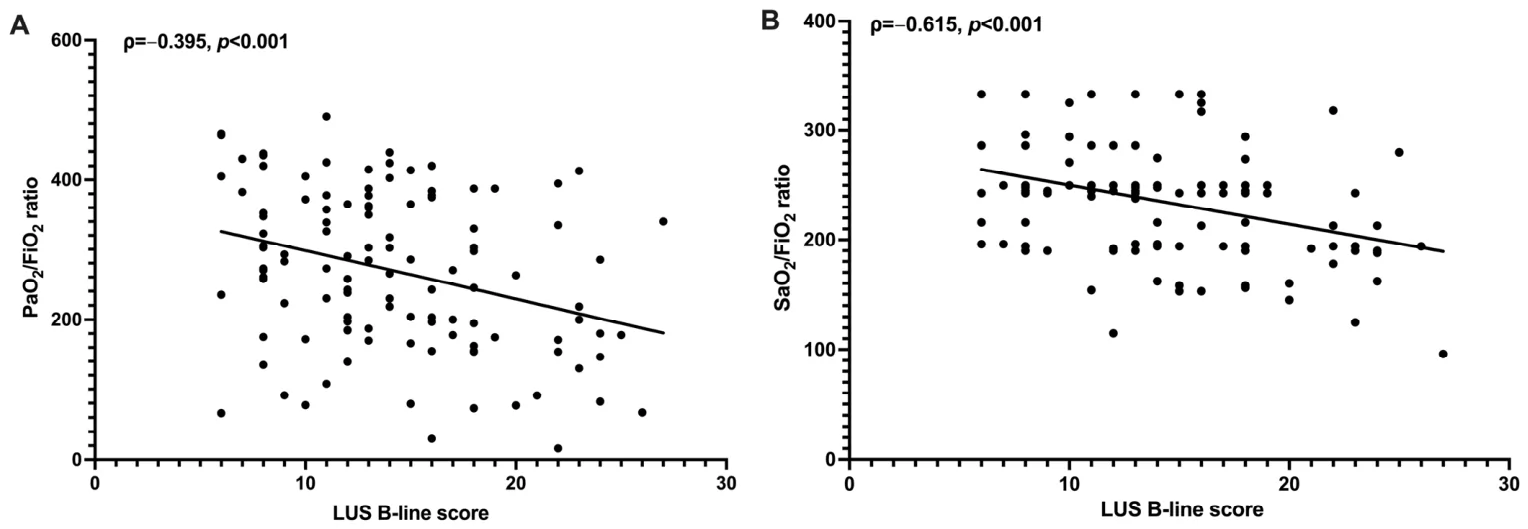
Correlation between LUS scores and postoperative oxygenation indices. Scatter plots showing the inverse correlations between admission LUS scores and postoperative PaO_2_/FiO_2_ (**A**) and SpO_2_/FiO_2_ (**B**). Higher LUS scores were associated with poorer postoperative oxygenation, supporting the value of LUS as an indicator of early postoperative pulmonary aeration loss and impaired gas exchange.

Spearman correlation analysis further demonstrated an inverse relationship between LUS scores and gas-exchange efficiency, with a moderate correlation for PaO_2_/FiO_2_ (⍴ = −0.395, *p* < 0.001) and a stronger correlation for SpO_2_/FiO_2_ (⍴ = −0.615, *p* < 0.001). Scatter plots in Figure 3 visually depict this monotonic decline in oxygenation with expanding B-line extent.

### 3.4. Risk Factors for PMV

Univariate logistic regression identified high LUS scores as a significant risk factor for PMV (OR = 1.383, 95% CI = 1.115–1.562, *p* < 0.001), along with MELD score, intraoperative fluid balance, surgical duration, oxygenation indices, PEEP and APACHE II scores (Table 3). In the multivariable model, MELD score (OR: 1.118; 95% CI: 1.043–1.198, *p* = 0.00), intraoperative fluid balance (OR: 1.329, 95% CI: 1.030–1.714, *p* = 0.03), postoperative oxygenation index (OR: 0.992, 95% CI: 0.986–0.998, *p* = 0.02) and LUS score (OR: 1.551, 95% CI: 1.265–1.901, *p* < 0.001) remained independently associated with PMV. No significant multicollinearity was detected among the included variables (all VIFs < 5), and the model demonstrated good calibration (Hosmer–Lemeshow χ^2^ = 8.039, *p* = 0.43).

### 3.5. Predictive Performance of LUS and the Multivariable Model

Interobserver agreement was excellent, with a kappa coefficient of 0.84 (95% CI: 0.61–1.00) for regional scores and an ICC of 0.92 (95% CI: 0.85–0.96) for total LUS score.

**Figure 4:**
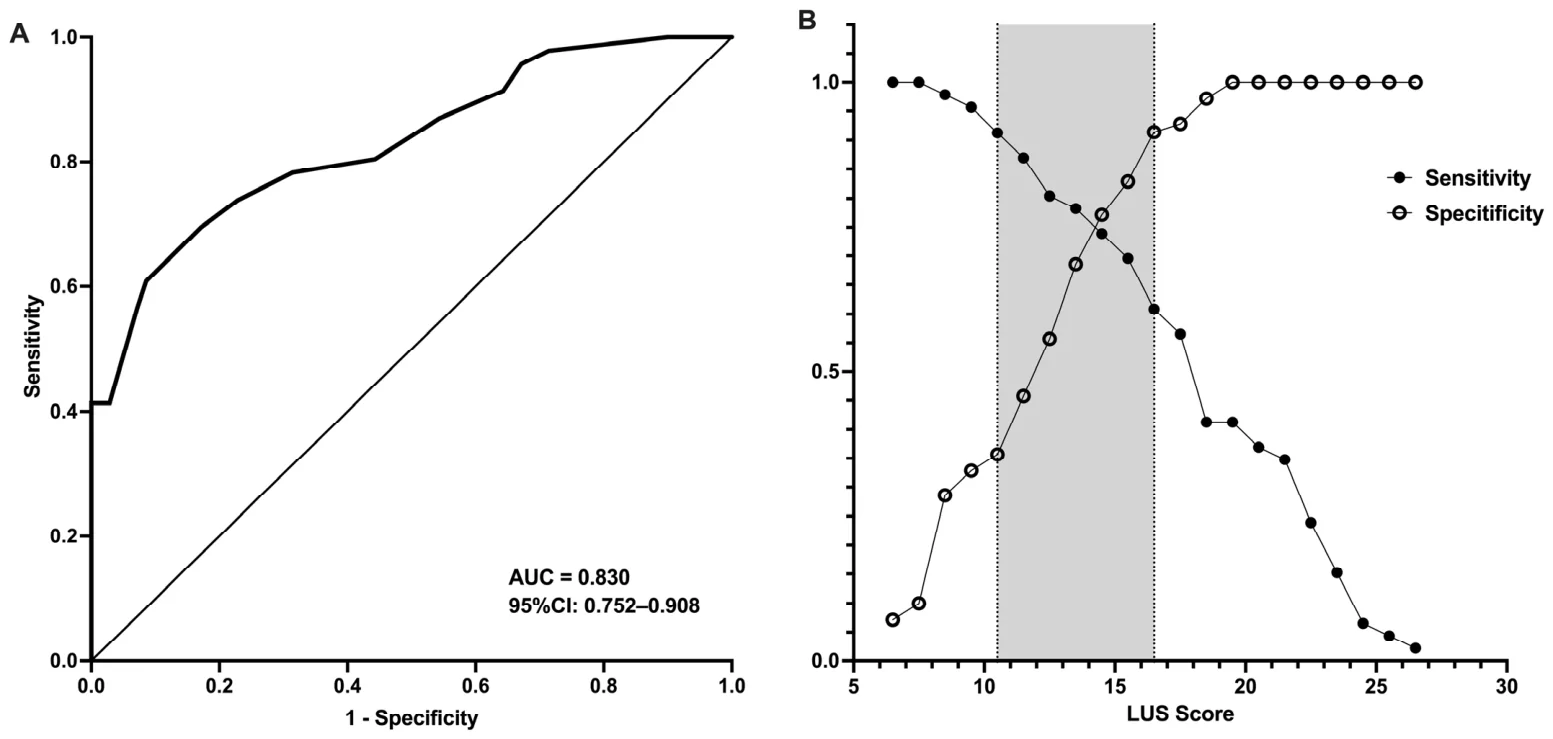
Predictive performance of LUS score for PMV. (**A**) ROC curve showing the discriminative performance of the LUS score for identifying patients at risk of PMV (AUC = 0.830). (**B**) Sensitivity–specificity crossover plot used to determine the optimal cutoff value for the LUS score. The shaded area represents the threshold range with the most balanced sensitivity and specificity.

ROC curve analysis demonstrated good discrimination of the LUS score for PMV, with an AUC of 0.830 (95% CI: 0.752–0.908). An optimal cutoff of 15.5 points provided 70% sensitivity and 83% specificity (Figure 4A). An inconclusive zone based on 90% sensitivity and specificity thresholds identified LUS scores >16.5 as indicating a high likelihood of prolonged postoperative ventilatory support (Figure 4B).

**Figure 5:**
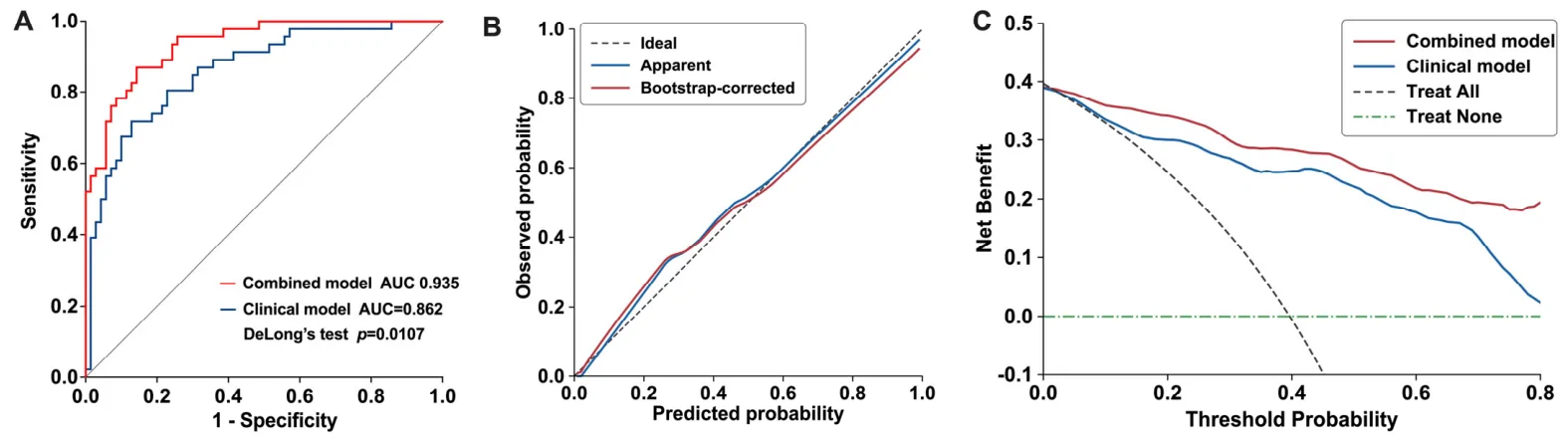
Predictive performance, calibration, and clinical utility of the integrative model for prolonged mechanical ventilation. (**A**) ROC curves’ comparison comparing the integrative model with the baseline clinical model. The addition of LUS scores improved model discrimination, with an AUC of 0.935 vs. 0.862 (DeLong test, *p* = 0.0107). (**B**) Bootstrap calibration plot showing the agreement between predicted probabilities and observed outcomes for the integrative model. The bias-corrected calibration curve closely followed the ideal reference line. (**C**) DCA demonstrating that the integrative model provided greater net clinical benefit than the baseline clinical model across a range of clinically relevant threshold probabilities.

To evaluate the incremental value of LUS for early postoperative risk stratification, hierarchical models were constructed according to the temporal availability of predictors. The apparent AUC increased from 0.758 (95% CI: 0.664–0.851) for the preoperative model including MELD score to 0.800 (95% CI: 0.716–0.884) after addition of intraoperative fluid balance and to 0.862 (95% CI: 0.793–0.931) after further incorporation of postoperative PaO_2_/FiO_2_. After postoperative PaO_2_/FiO_2_ had been incorporated into Model 3, addition of the LUS score (Model 4) further increased the AUC to 0.935 (95% CI: 0.894–0.976), corresponding to an absolute increase of 0.073 (DeLong *p* = 0.011) (Table 4, Figure 5A). The apparent Brier score decreased from 0.146 for Model 3 to 0.102 for Model 4.

Internal validation using 1000 bootstrap resamples yielded an optimism-corrected AUC of 0.921 and an optimism-corrected Brier score of 0.118 for the final model. The optimism-corrected calibration intercept and slope were −0.055 and 0.851, respectively, indicating some degree of model optimism. Consistent with this finding, bootstrap-based uniform coefficient shrinkage using 1000 resamples yielded a shrinkage factor of 0.856, corresponding to approximately 14% shrinkage of the original regression coefficients. The bootstrap-corrected calibration curve nevertheless showed reasonable agreement between predicted and observed probabilities (Figure 5B). Decision curve analysis demonstrated greater net benefit for the model incorporating LUS than for the clinical–physiological model without LUS across threshold probabilities (0.1–0.5) (Figure 5C).

### 3.6. Sensitivity Analysis

In a sensitivity analysis using mechanical ventilation >48 h as an alternative outcome, 29 of 116 patients (25%) met the endpoint. In Firth’s penalized logistic regression adjusting for MELD score, intraoperative fluid balance, postoperative PaO_2_/FiO_2_, and LUS score, LUS remained independently associated with prolonged ventilation (OR = 1.197, 95% CI: 1.084–1.336, *p* < 0.001). The LUS score also retained good discrimination for ventilation >48 h (AUC = 0.788, 95% CI: 0.692–0.884).

## 4. Discussion

Pulmonary complications, including pulmonary edema, ARDS, and infections, remain critical challenges after liver transplantation, frequently resulting in PMV, impaired gas exchange, and prolonged ICU and hospital stays [4,13,14]. Early postoperative risk stratification for PMV is critical for optimizing perioperative respiratory management and improving patient outcomes.

Recent studies have explored the prognostic value of perioperative LUS in different surgical populations. Rucham et al. demonstrated that LUS scores increased after elective intra-abdominal surgery, although these changes were not significantly associated with clinically relevant postoperative pulmonary complications in a relatively low-risk population [15]. In contrast, Diwakar et al. reported that an ultrasound-based extravascular lung water score after pediatric cardiac surgery was independently associated with time to extubation [16]. These findings suggest that the prognostic relevance of postoperative LUS may vary according to surgical population and baseline pulmonary risk. In this prospective cohort of 116 liver transplant recipients, early postoperative LUS scores experienced longer MV duration and ICU stay. LUS alone demonstrated good discriminative ability for PMV (AUC = 0.830, 95% CI: 0.752–0.908), with an optimal cutoff of ≥15.5 yielding 70% sensitivity and 83% specificity. These findings are consistent with previous reports in general postoperative populations [8,10], although the higher threshold observed in our cohort likely reflects increased baseline pulmonary vulnerability among liver transplant recipients. Spearman correlation analysis demonstrated an inverse relationship between LUS scores and oxygenation indices (PaO_2_/FiO_2_ ⍴ = −0.395; SpO_2_/FiO_2_ ⍴ = −0.615; both *p* < 0.001), supporting the physiological relevance of LUS-derived aeration assessment.

Mechanistically, LUS provides an estimate of lung aeration loss by reflecting the balance between extravascular lung water and alveolar air content [17,18]. Pulmonary edema following liver transplant may arise from hydrostatic mechanisms, such as fluid overload or elevated cardiac filling pressures [19,20], as well as non-hydrostatic processes related to increased pulmonary permeability associated with end-stage liver disease, ischemia–reperfusion injury, transfusion reactions, or surgical stress [4,21,22]. Severe loss of lung aeration may progress to gravity-dependent peripheral consolidation. Although postoperative oxygenation indices and LUS are both related to postoperative pulmonary dysfunction, they represent complementary rather than identical aspects of respiratory status. LUS primarily reflects structural alterations in pulmonary aeration, including interstitial abnormalities and peripheral consolidations extending to the pleural surface, whereas PaO_2_/FiO_2_ reflects the functional consequences of impaired gas exchange. Therefore, despite their biological association, these variables capture complementary aspects of postoperative respiratory status and may provide additional information for risk stratification when interpreted together. Consistent with these mechanisms, our multivariable analysis identified LUS score (OR = 1.551, 95% CI: 1.265–1.901, *p* < 0.001), MELD score, intraoperative fluid balance, and postoperative oxygenation index remaining independently associated with PMV. Importantly, incorporation of LUS into a multivariable model significantly improved discrimination (AUC = 0.935, 95% CI: 0.894–0.976; DeLong test, *p* = 0.011), while DCA demonstrated greater net benefit across clinically relevant threshold probabilities. The prognostic target in the present study was subsequent prolonged mechanical ventilation, rather than the future development of pulmonary dysfunction itself. Accordingly, early postoperative LUS should be viewed as a means of identifying and quantifying pulmonary aeration abnormalities already present in the immediate postoperative period that may carry prognostic information regarding subsequent ventilatory support. However, the statistical net benefit observed in DCA should not be interpreted as evidence that LUS-guided management improves clinical outcomes.

From a hypothesis-generating perspective, early identification of patients at higher risk of PMV using LUS may provide a basis for future studies evaluating individualized postoperative management strategies. Potential approaches for prospective evaluation could include individualized fluid management, lung-protective ventilation and recruitment strategies when appropriate, optimization of extubation timing, and early respiratory physiotherapy. However, none of these LUS-guided interventions were evaluated in the present study, and the current findings should not be interpreted as evidence supporting their effectiveness. Whether management guided by LUS-based risk stratification can improve ventilator-related or other postoperative outcomes requires confirmation in prospective interventional studies.

It is worth noting that the overall LUS score integrates multiple ultrasound findings, including B-lines, pleural abnormalities, and peripheral consolidations, each of which may reflect distinct pathophysiological mechanisms [23]. While B-lines are generally associated with interstitial edema or increased extravascular lung water, peripheral consolidations may indicate atelectasis, inflammatory lung injury, or postoperative pneumonia [24]. Therefore, the aggregate LUS score should be interpreted as a comprehensive marker of postoperative pulmonary aeration loss rather than a disease-specific indicator [25]. Future studies evaluating the prognostic contribution of individual ultrasound patterns may further improve mechanistic understanding and refine postoperative respiratory risk stratification.

Compared with conventional chest radiography, LUS offers several practical advantages in the postoperative setting. Conventional radiographs may fail to detect early or subtle pulmonary changes, whereas LUS allows dynamic bedside visualization of B-lines, peripheral consolidation, and pleural effusions. LUS can be safely repeated in hemodynamically unstable patients or those unable to undergo routine imaging [26,27]. Furthermore, integration with cardiac ultrasound parameters (E/A and E/Ea ratios) may provide additional information when evaluating the potential hemodynamic contribution to postoperative pulmonary abnormalities and may inform individualized assessment of fluid status [28].

Methodologically, we applied a standardized 12-region LUS protocol [29,30], to comprehensively assess anterior, lateral, and posterior lung zones. Although simplified six-point or BLUE protocol-based approaches may enable rapid screening, omission of posterior regions may lead to underestimation of lung pathology [23,26]. LUS scoring was performed by trained operators, and interobserver agreement was high (κ = 0.838, 95% CI: 0.610–1.000), supporting methodological reliability. Nevertheless, although a standardized scanning and scoring protocol was used and interobserver agreement was high, ultrasound operators were not fully blinded to contemporaneous clinical and ventilator information. Therefore, the observer’s influence on image acquisition or interpretation cannot be excluded and represents a potential source of measurement bias.

Importantly, the proposed model was developed in adult primary liver transplant recipients without severe chronic pulmonary disease or preoperative mechanical ventilation. Its applicability should therefore be currently limited to similar clinical settings, and external validation in independent, multicenter cohorts is essential before the model can be considered for clinical implementation.

Several limitations should be considered. First, this was a single-center study with a relatively small number of PMV events, and exclusion of patients with severe chronic pulmonary disease may further limit generalizability. Although bootstrap internal validation and coefficient shrinkage were performed, the optimism-corrected calibration slope below one indicated some degree of model overfitting; therefore, external validation in independent multicenter cohorts is essential before clinical implementation. Second, LUS was performed 1–3 h after ICU admission while patients were still mechanically ventilated and had already undergone substantial perioperative hemodynamic and respiratory perturbations. Thus, LUS abnormalities may partly reflect pulmonary dysfunction already present in the immediate postoperative period, and our findings should be interpreted as supporting early postoperative risk stratification rather than pre-symptomatic prediction. Third, postoperative LUS and PaO_2_/FiO_2_ represent complementary but biologically related aspects of pulmonary dysfunction, and residual overlap between these measures cannot be completely excluded. Finally, the duration of postoperative mechanical ventilation may be influenced not only by pulmonary dysfunction but also by sedation, clinicians’ decision-making, and institutional extubation practices. Although the association between LUS and prolonged ventilation remained robust in the sensitivity analysis using a >48 h threshold, the endpoint should not be interpreted as a purely physiological measure. Future multicenter studies are needed to externally validate the model and determine whether LUS-guided strategies can improve postoperative outcomes.

## 5. Conclusions

Early postoperative LUS enables a rapid, non-invasive evaluation of lung aeration impairment in liver transplant recipients and is independently associated with PMV. Incorporating LUS into a multivariable framework with established clinical variables, including MELD score, intraoperative fluid balance, and early postoperative oxygenation, significantly improved model discrimination. This integrated approach may facilitate early identification of patients at higher risk of PMV and support postoperative risk stratification. Prospective multicenter studies are warranted to externally validate these findings and determine whether LUS-guided risk stratification can facilitate individualized postoperative respiratory management and improve clinical outcomes.

## Figures and Tables

**Table 1:** Baseline characteristics of patients according to invasive MV duration. Data were expressed as mean (SD), n/N (%), or median (IQR).

Variables	Ventilation < 24 h (*n* = 70)	Ventilation ≥ 24 h (*n* = 46)	*p*
Baseline characteristics			
Age (years)	51.4 (10.2)	53.3 (9.4)	0.33
Male (%)	53/70 (76%)	32/46 (70%)	0.46
BMI (kg/m^2^)	22.9 (20.0–25.6)	23.4 (21.6–26.8)	0.29
Smoking, *n* (%)	15/70 (21%)	7/46(15%)	0.40
Hypertension, *n* (%)	9/70 (13%)	7/46 (15%)	0.72
Diabetes, *n* (%)	8/70 (11%)	6/46 (13%)	0.80
Etiology of liver failure			
HBV, *n* (%)	29/70 (48%)	19/46 (41%)	0.52
Autoimmune liver disease, *n* (%)	9/70 (13%)	4/46 (8.7%)	0.49
Alcoholic liver disease, *n* (%)	8/70 (10%)	5/46 (11%)	0.88
Clinical characteristics			
MELD score	12.7 (8.2–18.4)	22.5 (14.8–29.5)	<0.001
Duration of surgery (h)	9.7 (2.8)	11.7 (2.6)	<0.001
Intraoperative fluid balance (L)	4.7 (2.4–6.4)	5.8 (4.1–9.0)	0.002
Postoperative characteristic			
PaO_2_/FiO_2_ (mmHg)	303.8 (233.7–384.9)	189.8 (142.4–298.8)	<0.001
SpO_2_/FiO_2_	249.0 (216.0–286.0)	203.5 (185.5–243.5)	<0.001
Requirement for vasopressor, *n* (%)	26/70 (43%)	15/46 (33%)	0.29
Lactate (mmol/L)	2.3 (1.4, 4.3)	2.8 (1.9, 5.3)	0.16
PEEP (cmH_2_O)	5.0 (5.0–5.0)	5.5 (5.0–8.0)	<0.001
Tidal volume (mL)	450 (400–480)	450 (400–480)	0.72
APACHE II	11.0 (8.8–14.0)	15.0 (12.0–21.5)	<0.001
Prognosis			
Duration of ICU stay (day)	3.0 (2.0–4.0)	10.0 (5.8–22.3)	<0.001
Duration of hospitalization (day)	28.0 (21.8–36.3)	37.0 (23.8–53.8)	0.015
In-hospital mortality, *n* (%)	2/70(3%)	7/46(15%)	0.015
Ultrasound parameters			
LUS score	12.0 (8.0–14.0)	18.0 (14.0–22.3)	<0.001
LVOT VTI	20.6 (2.9)	20.6 (3.8)	0.94
E/A ratio	1.0 (0.8–1.9)	1.3 (0.9–1.7)	0.015
E/Ea ratio	8.4 (7.6–10.1)	8.7 (7.4–11.1)	0.68

Selected major etiologies of underlying liver disease are presented. BMI: Body mass index; HBV: hepatitis B virus; MELD: model for end-stage liver disease; PaO_2_/FiO_2_: partial pressure of oxygen/fraction of inspiration oxygen; SpO_2_/FiO_2_: pulse oximetry oxygen saturation/fraction of inspired oxygen; PEEP: positive end-expiratory pressure; APACHE II: Acute Physiology and Chronic Health Evaluation II; LUS, lung ultrasound; LVOT: left ventricular outflow tract; VTI: velocity–time integral; E: peak velocities of early fillings; A: peak velocities of late fillings; Ea: early diastolic velocity of mitral annular displacement.

**Table 2:** Baseline characteristics and short-term outcomes across lung ultrasound score tertiles.

Variables	Low LUS (*n* = 38) LUS (0–11)	Intermediate LUS (*n* = 34) LUS (12–15)	High LUS (*n* = 44) LUS (16–36)	*p*
Age (years)	52.8 (8.7)	49.1 (11.3)	54.0 (9.2)	0.08
Male (%)	28/38 (74%)	24/34 (71%)	33/44 (75%)	0.91
BMI (kg/m^2^)	22.4 (19.8–25.3)	23.3 (20.2–27.5)	23.5 (21.2–26.2)	0.30
MELD score	15.8 (8.3–22.8)	13.6 (10.4–20.8)	18.7 (11.9–24.3)	0.32
Duration of surgery (h)	10.2 (3.0)	9.8 (2.6)	11.3 (2.9)	0.05
Intraoperative fluid balance (L)	4.7 (2.4–6.5)	5.3 (3.0–6.7)	5.3 (3.0–8.1)	0.37
Requirement for vasopressor, *n* (%)	17/38 (45%)	16/34 (47%)	24/44 (55%)	0.71
PaO_2_/FiO_2_ (mmHg)	313.9 (233.8–405.0)	284.7 (202.0–365.0)	199.9 (154.2–333.8)	0.005
SpO_2_/FiO_2_	250.0 (242.3–286.0)	243 (193.5–277.8)	213.0 (190.0–250.0)	0.006
APACHE II	11.0 (9.0–14.5)	11.5 (8.0–15.3)	15.0 (11.0–20.8)	0.002
Duration of mechanical ventilation (h)	12.0 (9.0–17.0)	13.0 (9.0–20.0)	34.0 (13.5–80.5)	<0.001
Duration of ICU stay (day)	3.0 (2.0–4.0)	4.0 (3.0–6.3)	6.5 (4.0–15.8)	<0.001
Duration of hospitalization (day)	30.0 (21.0–45.8)	28.0 (23.0–35.3)	35.0 (24.3–54.3)	0.12

**Table 3:** Logistic regression analysis for the prediction of prolonged MV.

Variables	Univariate Analysis			Multivariate Analysis		
	OR	95% CI	*p*	OR	95% CI	*p*
Age	1.019	0.981–1.059	0.32			
BMI	1.089	0.964–1.230	0.17			
MELD score	1.098	1.046–1.152	<0.001	1.118	1.043–1.198	0.002
Intraoperative fluid balance	1.305	1.129–1.509	<0.001	1.329	1.030–1.714	0.03
Duration of surgery	1.330	1.129–1.568	0.001	1.011	0.777–1.315	0.94
PaO_2_/FiO_2_	0.991	0.987–0.995	<0.001	0.992	0.986–0.998	0.02
SpO_2_/FiO_2_	0.986	0.977–0.994	0.001			
PEEP	1.587	1.227–2.053	<0.001			
APACHE II	1.110	1.033–1.192	0.004	1.022	0.912–1.147	0.70
LUS score	1.383	1.225–1.562	<0.001	1.551	1.265–1.901	<0.001
E/A	1.446	0.625–3.346	0.39			

**Table 4:** Incremental performance of hierarchical models for prolonged mechanical ventilation.

Model	Predictors	AUC (95% CI)	Brier Score
Model 1: Preoperative	MELD score	0.758 (0.664–0.851)	
Model 2: Perioperative	Model 1 + intraoperative fluid balance	0.800 (0.716–0.884)	
Model 3: Clinical–physiological	Model 2 + postoperative PaO_2_/FiO_2_	0.862 (0.793–0.931)	0.146
Model 4: Clinical–physiological + LUS	Model 3 + LUS score	0.935 (0.894–0.976)	0.102

AUCs and Brier scores presented in this table represent apparent model performance; bootstrap-corrected performance of the final model is reported separately in the text.

## Data Availability

The datasets generated or analyzed during the current study are available from the corresponding authors on reasonable request.
